# Quantifying the dilution of the radiocesium contamination in Fukushima coastal river sediment (2011–2015)

**DOI:** 10.1038/srep34828

**Published:** 2016-10-03

**Authors:** Olivier Evrard, J. Patrick Laceby, Yuichi Onda, Yoshifumi Wakiyama, Hugo Jaegler, Irène Lefèvre

**Affiliations:** 1 Laboratoire des Sciences du Climat et de l’Environnement (LSCE/IPSL), Unité Mixte de Recherche 8212 (CEA/CNRS/UVSQ), Université Paris-Saclay, Gif-sur-Yvette (France); 2 Center for Research in Isotopes and Environmental Dynamics (CRIED), University of Tsukuba, Tsukuba (Japan); 3 Institute of Environmental Radioactivity (IER), University of Fukushima, Fukushima (Japan)

## Abstract

Fallout from the Fukushima Dai-ichi nuclear power plant accident resulted in a 3000-km^2^ radioactive contamination plume. Here, we model the progressive dilution of the radiocesium contamination in 327 sediment samples from two neighboring catchments with different timing of soil decontamination. Overall, we demonstrate that there has been a ~90% decrease of the contribution of upstream contaminated soils to sediment transiting the coastal plains between 2012 (median – M – contribution of 73%, mean absolute deviation – MAD – of 27%) and 2015 (M 9%, MAD 6%). The occurrence of typhoons and the progress of decontamination in different tributaries of the Niida River resulted in temporary increases in local contamination. However, the much lower contribution of upstream contaminated soils to coastal plain sediment in November 2015 demonstrates that the source of the easily erodible, contaminated material has potentially been removed by decontamination, diluted by subsoils, or eroded and transported to the Pacific Ocean.

Large quantities of radiocesium were deposited on soils of Fukushima Prefecture following the Fukushima Dai-ichi Nuclear Power Plant (FDNPP) accident in March 2011^1^. Approximately 66% of high levels of radioactive fallout (>1000 kBq m^−2^) took place over a mixture of deciduous and evergreen forests^2^. However, soil erosion is unlikely to occur in forested landscapes in the Fukushima region because of the accumulation of a thick litter layer of organic matter protecting the soil from rainfall driven soil erosion^3^,^4^. Radiocesium was shown to strongly bind to the fine soil particles^5^ that are preferentially mobilized and transported by overland flow to river systems^6^. This transfer of fine sediments, and their bound radiocesium, is exacerbated in rice paddy fields that are directly connected to rivers with irrigation systems^7^. The radiocesium contamination of paddy fields is concentrated in the uppermost 2–5 cm of the soil where it is readily available for soil erosion^8^,^9^. Accordingly, paddy fields were demonstrated to be a major source of particle-bound radiocesium to rivers in this region^4^,^10^.

Since 2012, Japanese authorities have made considerable progress decontaminating paddy fields and rural residential areas^11^. In heavily contaminated areas, remediation consists of removing the vegetation and replacing the topsoil (~5 cm) with a new substrate. There is a need to investigate whether these operations impact radiocesium contamination levels measured in sediment transiting the rivers in this region. In particular, it is important to examine the efficacy of this extensive decontamination effort (e.g. an estimated 1–16 trillion yen (~10–140 billion USD) for a target area of 9000 km^2 ^11).

Radiocesium has been widely used to trace surface and subsurface source contributions to river sediment^12^,^13^,^14^. Currently, a significant proportion of radiocesium deposited in paddy fields has been removed by decontamination^15^,^16^. Decontaminated areas now have low radiocesium levels in comparison to contaminated areas. These decontaminated landscapes have become a potential source of radiocesium depleted sediments, similarly to subsurface sources. These depleted sources could therefore be modelled, as one source end-member, with the contaminated surface sources (with high radiocesium activities), being the other end member, to quantify the dilution of radiocesium contamination in sediment transiting rivers draining the main plume of fallout from the FDNPP accident.

Accordingly, a modified sediment tracing technique is used to quantify the relative contributions of contaminated and depleted radiocesium sediment sources in two paired catchments in the Fukushima region. The paired catchments include the Niida catchment (275 km^2^), where decontamination started in 2013, and the neighboring Mano catchment (175 km^2^), where decontamination works started in 2014–2015. The only significant difference between these catchments is a major dam located between the upstream – more contaminated – section of the Mano catchment and the coastal plain – where soils contain very low radiocesium concentrations (Fig. 1). In contrast, the only major dam in the Niida catchment is situated on a tributary and it will therefore have a limited impact on sediment transport in the main river stem.

Due to the proximity of the Niida and Mano catchments, we hypothesize that they are exposed to similar climatic conditions and therefore potential differences in sediment radiocesium contamination in their upper reaches will reflect the different timing of the implementation of the remediation works. The validity of this hypothesis is supported by the fact that initial radiocesium soil contamination levels were the highest in upper parts of both catchments and lowest in their coastal plains. Furthermore, both catchments were shown to receive similar rainfall erosivity with similar spatial distributions of rainfall during major typhoon events^17^. Distribution models were used to quantify the relative contributions from the two end-members (i.e. contaminated and depleted sources) to sediment in these two catchments at 41 sites that were sampled every 6 months from November 2011 to November 2015.

## Results

### Spatial patterns of decontamination works in the Niida and the Mano catchments

Remediation started late in 2013 in the headwaters of the Niida catchment, along the main stream of the main Niida tributary (Fig. 2). Thereafter, the decontamination works moved progressively downstream along this tributary. Decontamination expanded along the Iitoi River, the central tributary of the Niida, in 2014. In the southern tributary, the Hiso River, the majority of the decontamination started in 2015.

In the Mano catchment, remediation works started in late 2014. As remediation works started only in late 2014 in this catchment, variations in initial contamination proportions observed in upper parts of the Mano catchment between autumn 2011 and spring 2014 should reflect variations in erosion intensity and sediment supply due to heavy rainfall, rather than soil erosion that may have been accelerated during initial decontamination operations.

### Evolution of contamination levels in the Niida River

Contamination of sediment transiting the Niida River decreased from 2011 (median – M – 65% of contribution from the upstream contaminated soils) to 2015 (M 10%; Fig. 3). The progressive transfer of contaminated material from the upper catchment to the coastal plain – where it may be stored temporarily in the channel – is reflected by the higher contribution of upstream soils recorded in the downstream river sections in autumn 2013 (M 41%, median absolute deviation - MAD 22%) and in spring 2015 (M 37%, MAD 28%). After a first flush of contamination during the year that followed the accident (from a maximum median contribution of upstream soils of 40% to a minimum of 16%, in spring 2012 (see Fig. 3)), results of the models indicate the occurrence of a succession of increases (autumn 2013 and spring 2015, maximum M 41%) and decreases (spring 2014, M 13% and autumn 2015, minimum M 10%) of these upper catchment soil contributions in both upstream and downstream river reaches.

### Evolution of contamination levels in the Mano River

The sediment disconnectivity induced by the Mano Dam (with the exception of a major dam release that occurred in 2011) provides an opportunistic method to estimate the decrease in upstream contaminated soil median contributions (from 68% to 6%) that would have been observed in the coastal plains of the Niida catchment in the absence of continuous supply of contaminated material originating from upper catchment parts (Fig. 4). After spring 2012, the median contribution of these upstream contaminated soils remained systematically below 20% in the coastal plains, although two low-magnitude peaks of contamination were observed in autumn 2013 (M 16%) and in spring 2015 (M 10%; Fig. 4). When comparing the Niida and Mano contamination level evolution, it becomes very apparent that a large quantity of contaminated sediment is likely stored in the Mano Dam reservoir.

### Evolution of contamination levels in sediment collected in upstream tributaries of the Niida River

When the model results are grouped for each upstream tributary of the main Niida River, the evolution of the contaminated soil contribution is different than what was observed for the entire upstream Niida catchment (Fig. 5A). The median contribution of contaminated soils to sediment increases from the north to the south (from a median maximum of 69% in the upstream Niida mainstream to 98% in the Hiso River), reflecting the spatial pattern of the main radioactive pollution plume. Peaks of contaminated soil contributions are observed during spring 2014 (M 99% in the Iitoi River) and spring 2015 (M 98% in the Hiso River; M 63% in the Niida River). These peaks occurred after the majority of remediation works in paddy fields connected to these rivers was completed. For both the Niida and Iitoi Rivers, this peak in contamination is followed by a sharp decrease of the contribution of contaminated soils to sediment and their associated uncertainties.

## Discussion

These results illustrate the strong spatial and temporal variability of radioactive contamination levels of sediment transiting the Niida and the Mano Rivers between 2011 and 2015. This is likely explained by the occurrence of various erosion processes in the catchment and the collection of sediment deposits in different hydro-sedimentary contexts. Despite these spatial differences and the uncertainties associated with the model results, there is a major decrease in sediment contamination levels during this period. This trend is relevant with other observations reported in the literature^6^,^18^,^19^.

In particular, during the first two years that followed the nuclear accident, it was estimated that 1–5% of the initial catchment radiocesium inventory was exported by Fukushima coastal rivers to the Pacific Ocean^19^,^20^. This trend corresponds to the sharp decrease in the proportion of contaminated soil contribution modelled in sediment transiting the Niida and the Mano Rivers in this study. This observation is consistent with the typical radionuclide wash-off behaviour expected after a nuclear accident, with a short term intense ‘leaching’ phase during the weeks and months following the radionuclide emissions^21^,^22^.

Thereafter, the export of radionuclides bound to sediment is much slower, and should not exceed 1% of the initial radiocesium inventories in soils according to the estimations available from the literature^6^. Our modelling indicates that the decrease in the contribution of contaminated soil material found in sediment slowed down from 2012 onwards. However, in contradiction with the theoretical models describing the radionuclide wash-off with a negative exponential form, our results show the periodic increase in contamination levels following heavy flooding events, such as typhoons or spring floods (Table 1).

There were sediment-bound radiocesium peaks evident that were directly related to decontamination works. Although this increase is associated with large uncertainties, our modelling results suggest that remediation efforts may lead to a temporary increase in these levels during or shortly after the start of these operations. This may be explained by the fact that vegetation is removed to prepare these works^11^, which leaves the soils unprotected during heavy rainfall and enhances the production of overland flow. This observation had already been made in upper parts of the Niida River^16^. The large uncertainties may be explained by the integration of recently eroded soils originating from the remediated fields with material transiting in the river. However, when the investigation is prolonged during several seasons following the start of the remediation works, contamination levels in nearby river sediment decrease sharply, illustrating the removal of the easily erodible radiocesium source. According to the model results, decontamination is therefore likely efficient over the medium term, once areas have been completely remediated. Indeed, the uncertainties associated with the modelled contributions of contaminated soils to sediment decrease with time, which likely reflects the export of the most contaminated material to the Pacific Ocean and the progressive homogenization of the contamination level of the material transiting these coastal rivers.

In general, Fukushima river systems appear to be very reactive to both rainfall events (Table 1) and the radiocesium remediation (Fig. 5). This behaviour is likely explained by the high sediment connectivity of paddy fields, which were shown to be the main sources of contamination to the rivers shortly after the accident^23^. Transfers of radiocesium-bound sediment from paddy fields are exacerbated during different periods of the year, depending on the rice cultivation cycle. For instance, the puddling period, when the paddy soil is mixed with irrigation water down to a depth of 15 cm, is a very erosive period^24^. Together with the occurrence of a flood in early March in 2015 (Fig. 6), this probably explains the high contributions of contaminated soils modelled in sediment collected in spring 2015. In addition, runoff during typhoons that may occur at the end of the cultivation period may also trigger export of contaminated soil from paddies, as modelled in autumn 2013.

The very low proportions of contaminated soil median contributions modelled in sediment collected in November 2015 (6% in the Mano coastal plains vs. 10% in the Niida) indicate that the most erodible contaminated soils have already been flushed away to the Pacific Ocean. Although contaminated material is likely stored for a long time in the major dam in the Mano River catchment, the supply of contaminated sediment is more and more unlikely in catchments where remediation works have been completed.

In future, the potential contribution of forests as a perennial low-magnitude source of radioactive contamination to the river systems should be investigated, as forests cover two-thirds of the surface area in this region. Although both subsurface sources were also modeled as a depleted radiocesium source owing to the paired catchment design, the relative contributions of subsurface sources and decontaminated sources should be examined through the application of a different suite of appropriate fingerprints.

## Methods

### Study site

**Figure 1:**
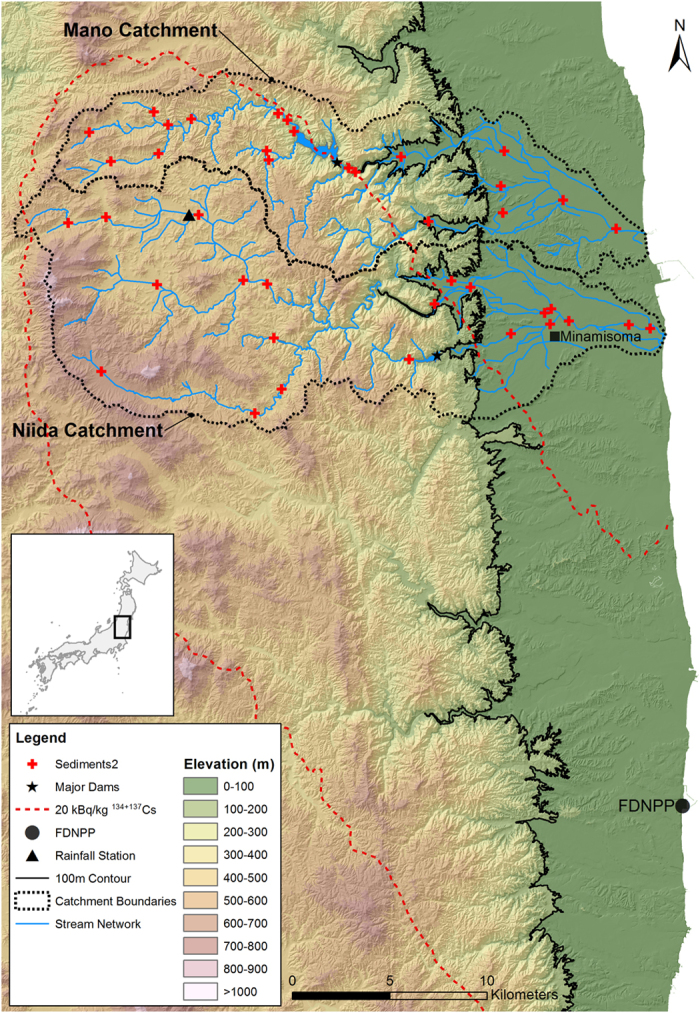
Elevation map of the investigated catchments within Fukushima Prefecture in Northern Japan (based on the Digital Elevation Model (DEM) with a 10 m resolution provided by the Geospatial Information Authority of Japan (GSI) from the Ministry of Land, Infrastructure, Transport and Tourism (http://www.gsi.go.jp/). Location of the main dams and the continuous rainfall monitoring station (Japanese Meteorological Agency, 2014). Initial radiocesium contamination contour lines were derived from Chartin *et al.*^7^. This original map was created using ArcGIS 10.3 software (http://www.esri.com/software/arcgis/).

**Figure 2:**
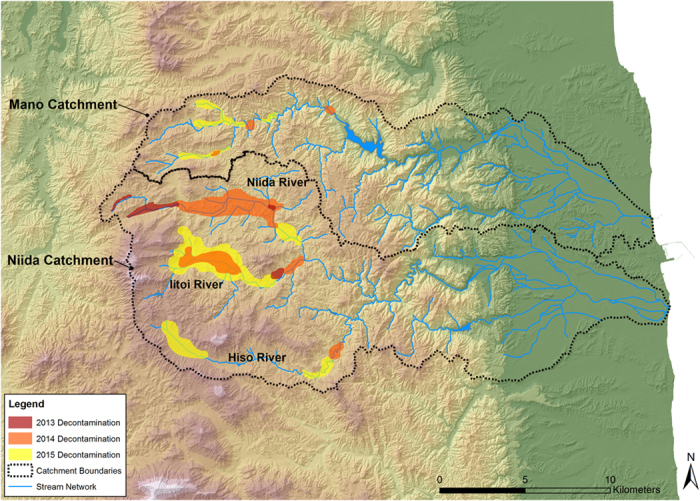
Progress of decontamination works in upper parts of the Niida catchment, from 2012 to 2015. This original map was created from field observations and a compilation of data available from the local Iitate Village municipality using ArcGIS 10.3 software (http://www.esri.com/software/arcgis/).

**Figure 3:**
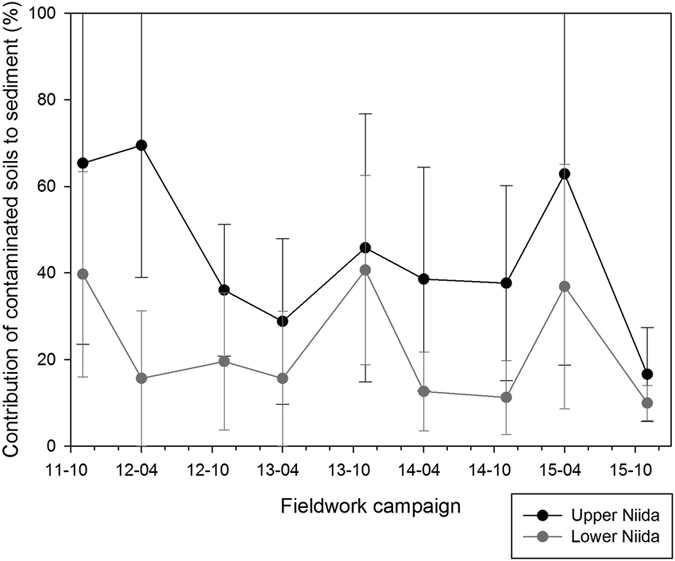
Median contribution and mean absolute deviation of upstream/contaminated soils to radiocesium content measured in sediment in the upper and lower Niida River sections, from November 2011 to November 2015. This original graph was created using SigmaPlot 12.5 software (http://www.sigmaplot.co.uk/products/sigmaplot/produpdates/prod-updates18.php).

**Figure 4:**
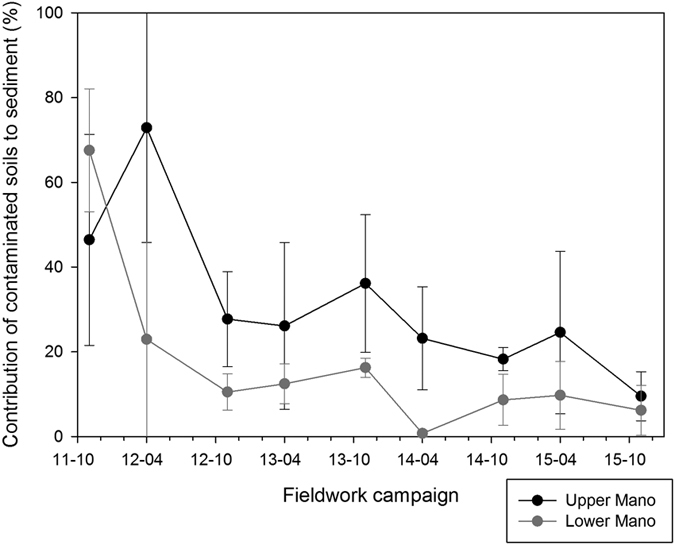
Median contribution and mean absolute deviation of upstream/contaminated soils to radiocesium content measured in sediment in the upper and lower Mano River sections, from November 2011 to November 2015. This original graph was created using SigmaPlot 12.5 software (http://www.sigmaplot.co.uk/products/sigmaplot/produpdates/prod-updates18.php).

**Figure 5:**
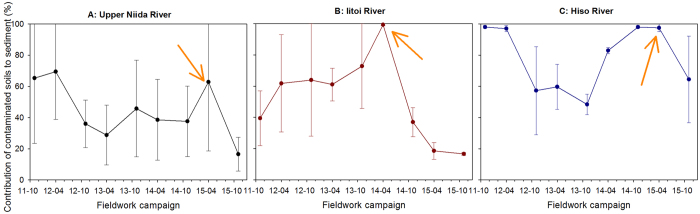
Median contribution and mean absolute deviation of contaminated soils to radiocesium content measured in sediment in the upstream tributaries of the Niida River, from November 2011 to November 2015. Arrows indicate the main peak of contamination modelled in rivers following the start of decontamination works along each tributary. This original graph was created using SigmaPlot 12.5 software (http://www.sigmaplot.co.uk/products/sigmaplot/produpdates/prod-updates18.php).

**Figure 6:**
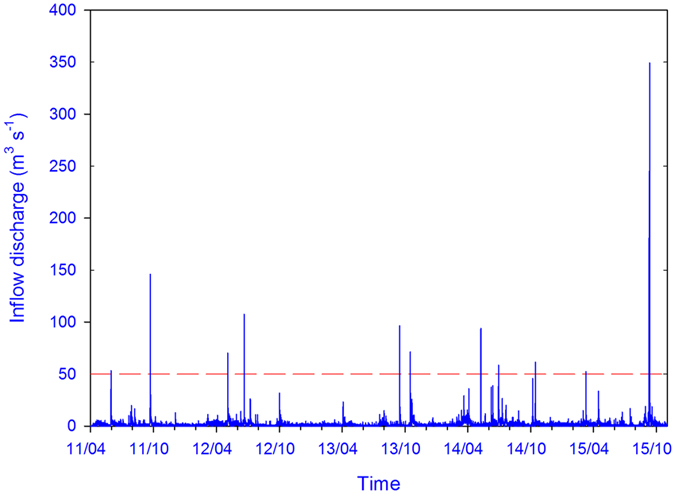
Evolution of Mano River discharge at the inlet of Mano Dam reservoir, from April 2011 to November 2015 (Fukushima Prefecture data). Red dashed horizontal line corresponds to a river discharge threshold of 50 m^3^ s^−1^ outlining the 10 highest peak discharge values recorded during the study period. This original graph was created using SigmaPlot 12.5 software (http://www.sigmaplot.co.uk/products/sigmaplot/produpdates/prod-updates18.php).

This research was conducted in the Niida (275 km^2^) and Mano (175 km^2^) catchments (Fig. 1). Catchment land use mainly consists of forest (75% SD 4%) and cropland (21% SD 4%)^10^. The main catchment features include an upstream coastal mountain range (<900 m) and a broad, more densely inhabited, coastal plain (i.e. <100 m) that occupies on average 25% (SD 6%) of both catchments. Soils in the upstream areas of these catchments were heavily contaminated, with soil radiocesium (^137^Cs) inventories ranging from 20 kBq kg^−1^ to 75 kBq kg^−1^. In contrast, soil radiocesium inventories in the lowland coastal plains were less than 20 kBq kg^−1 ^7. Maps showing the progress of remediation works in the study area were developed from field observations and a compilation of data available from the local municipality (Iitate Village) (Fig. 2).

Cumulative rainfall reached 5057 mm at a gauging station in the Niida catchment monitored between March 2011 and December 2015. This monitoring period included six typhoons and one tropical storm that resulted in >100 mm of rainfall. Mean annual rainfall in the Fukushima region is ~1400 mm^17^.

### Sampling

Two approaches were used to develop a ^137^Cs source dataset for modelling. First, soil samples (*n* = 160) were collected between 2011–2015 in locations reported to be highly connected to the stream network^7^. At each of these locations, ten subsamples (~5 g per subsample) were scraped from the soil surface randomly in a 10 m^2^ quadrant using a non-metallic trowel and composited into one sample. Second, ^137^Cs activities were included for 100 soil samples collected in these two catchments in June and July 2011 by the Japanese Ministry of Education, Culture, Sports, Science and Technology (MEXT)^10^. Sources samples collected in 2014–2015 from effectively decontaminated areas and the coastal plain region were used to develop source sample distributions (*n* = 44 in the Mano and *n* = 42 in the Niida catchment). Contaminated source sample distributions were derived from samples taken in the upstream areas (>100 m elevation Fig. 1) (n = 56 in the Mano and *n* = 118 in the Niida catchment).

Nine sediment sampling campaigns were conducted between November 2011 and November 2015. Sampling occurred bi-annually at 41 locations: in fall after the typhoon season, and in spring, after the snowmelt runoff. The goal was to sample deposited sediment that was transferred during these main erosive periods. In total, 327 sediment samples were obtained. Fine sediment samples were taken from material deposited after the last major event at the same sites, during each of the nine campaigns. These lag deposit samples were comprised of fine particulate material that settled on channel banks, inset benches and floodplains during the falling limb of the last significant hydro-sedimentary event. Ten subsamples (~5 g per subsample) of recently deposited material were taken with a plastic spatula over a 5 m reach and composited into one sample.

### Laboratory analyses

All samples were dried at 40 °C for ~48 h, sieved to 2 mm and ground to a fine powder in an agate mortar, and pressed into 15 mL polyethylene containers for measurement. ^137^Cs activities were determined with gamma spectrometry using coaxial N- and P-type HPGe detectors (Canberra/Ortec). ^137^Cs activities were measured at the 662 keV emission peak. More information on these methods can be found elsewhere^7^. All activities were decay-corrected to March 14, 2011, the date of the main radionuclide fallout deposition^25^.

### Distribution modelling

A distribution modelling approach was used to incorporate distributions throughout the entire modelling framework, including the source contribution terms^26^. Distribution models were used to quantify the relative contributions from two end-members, i.e. soils contaminated and depleted in radiocesium (^137^Cs). A summary of radionuclide activities characterizing both source types in the Niida and Mano catchments is provided in Table 2. A summary of the model results is given in Table 3.

For the two-source model, it is assumed that sediment samples constitute a discrete mixture of their sources, with the source contribution of A being *x*, and the contribution of B being 1−*x* where:


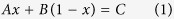


*C* is the in-stream sediment distribution, *A* and *B* are the two source distributions, and *x* is modelled as a truncated normal distribution (0 ≤ x ≤ 1) with a mixture mean (μ_m_) and standard deviation (σ_m_)^27^. The model is solved by minimizing the median difference between the distributions of both sides of Eq. 1 (i.e. *C* and *Ax* + *B* (1 − *x*)) with the Optquest algorithm in Oracle’s Crystal Ball software with a randomly generated mixture mean (*μ*_*m*_) and standard deviation (σ_m_).

The two source distributions were fit with the Crystal Ball software which selected the optimal distribution based on the results of three statistical tests: Anderson–Darling, Kolmogorov-Smirnov and Chi-Square. For the depleted source distributions in both catchments, log-normal distributions were modeled and for the contaminated sources, Maximum Extreme Distribution (also known as Gumbel distributions) were modeled.

Source contributions (x) were determined by simulating Eq. 1 with 2500 Latin Hypercube (500 bins) samples drawn from source and sediment distributions and varying the mixture mean (μ_m_) and standard deviation (σ_m_). This model simulation and solving process was then repeated 2500 times with the median proportional source contribution from these 2500 additional simulations, reported as the contribution of each source. Model uncertainty was calculated through summing three quantifiable model uncertainties: (1) the median absolute deviation of the individual source median contribution for the additional 2500 simulations; (2) the modelled standard deviation; and (3) the median absolute deviation of this modelled standard deviation for the 2500 model additional simulations^28^.

## Additional Information

**How to cite this article**: Evrard, O. *et al.* Quantifying the dilution of the radiocesium contamination in Fukushima coastal river sediment (2011–2015). *Sci. Rep.* **6**, 34828; doi: 10.1038/srep34828 (2016).

## Figures and Tables

**Table 1:** Characteristics of the main typhoons recorded between 2011–2015 for all rainfall stations within a 100 km radius of the FDNPP (modified from Laceby *et al.*)^17^.

Storm Name	Event classification	Period		Precipitation (mm)		
		Start	Finish	Mean	St. Dev.	Max
Songda	Typhoon	28/05/2011	29/05/2011	75	45	192
Ma-on	Typhoon	19/07/2011	20/07/2011	79	52	223
Talas	Severe tropical storm	31/08/2011	03/09/2011	63	52	225
Roke	Typhoon	19/09/2011	20/09/2011	231	48	339
Guchol	Typhoon	19/06/2012	19/06/2012	104	56	286
Jelawat	Typhoon	30/09/2012	30/09/2012	43	17	95
Toraji	Severe tropical storm	04/09/2013	05/09/2013	41	24	117
Man-yi	Typhoon	14/09/2013	15/09/2013	110	46	206
Wipha	Typhoon	15/10/2013	15/10/2013	113	18	151
Francisco	Typhoon	20/10/2013	24/10/2013	108	33	191
Mitag	Tropical storm	10/06/2014	11/06/2014	51	26	106
Neoguri	Typhoon	08/07/2014	09/07/2014	75	29	150
Halong	Typhoon	07/08/2014	10/08/2014	64	31	130
Phanfone	Typhoon	05/10/2014	05/10/2014	123	37	196
Vongfong	Typhoon	13/10/2014	13/10/2014	93	36	187
Nangka	Typhoon	15/07/2015	16/07/2015	85	45	191
Etau	Severe tropical storm	06/09/2015	09/09/2015	240	125	573

**Table 2:** Summary statistics of ^137^Cs (Bq kg^−1^) of source samples in the investigated catchments.

Catchment	Source	Mean	SD	Median	MAD
Niida (n = 160)	Contaminated source (n = 118)	31203	31737	24604	13401
	Depleted source (n = 42)	4635	3195	6483	1999
Mano (n = 100)	Contaminated source (n = 56)	21539	20179	16069	8114
	Depleted source (n = 44)	4417	7783	2580	1380

SD stands for Standard Deviation, and MAD stands for Median Absolute Deviation.

**Table 3:** Summary statistics of model results.

Proportion of initial soil radiocesium contamination modelled in sediment (%) – median (median absolute deviation)									
River reach	Field campaign								
	Nov. 2011	April 2012	Nov. 2012	April 2013	Nov. 2013	April 2014	Nov. 2014	April 2015	Nov. 2015
Mano catchment									
Upper Mano	46(25)	73(27)	28(11)	26(20)	36(16)	23(12)	18(3)	25(19)	9(6)
Lower Mano	68(14)	23(23)	10(4)	12(5)	16(2)	1(1)	9(6)	10(8)	6(6)
Niida catchment									
Upper Niida	65(42)	69(31)	36(15)	29(19)	46(31)	39(26)	38(23)	63(44)	17(11)
Iitoi	39(18)	62(31)	64(36)	61(10)	73(27)	99(1)	37(9)	19(5)	17(1)
Hiso	98(1)	97(2)	57(28)	60(15)	48(7)	83(2)	98(1)	98(3)	64(28)
Lower Niida	40(24)	16(16)	20(16)	16(16)	41(22)	13(9)	11(9)	37(28)	10(4)
